# Supplementary material for: Conditional chemoconnectomics (cCCTomics) as a strategy for efficient and conditional targeting of chemical transmission
Source: eLife. 2024 Apr 30;12:RP91927. doi: 10.7554/eLife.91927 (PMC11060718; doi:10.7554/eLife.91927)
Supplement: Figure 3—source data 3. [file elife-91927-fig3-data3.zip › Figure 3 source data3 part 1.zip!/Figure 3-Source data 3-part1/1071f3-1-CRISPResso_Report_jvi77nmx.zip!/CRISPResso_on_1071f3_4/1a.Read_barplot.pdf]

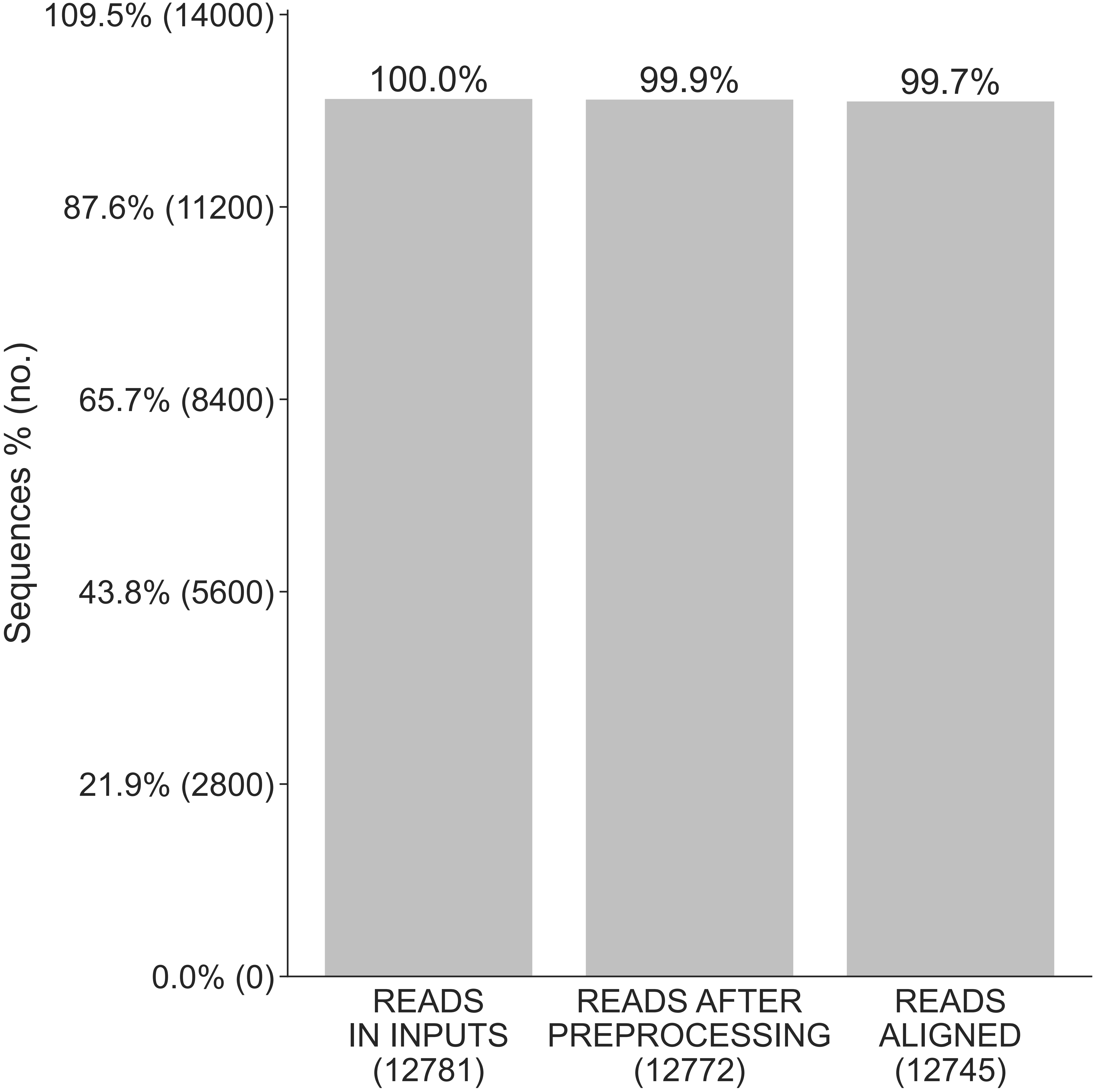

109.5% (14000)
87.6% (11200)
65.7% (8400)
43.8% (5600)
21.9% (2800)
0.0% (0)
Sequences % (no.)
100.0%
99.9%
99.7%
READS IN INPUTS (12781)
READS AFTER PREPROCESSING (12772)
READS ALIGNED (12745)
